# Differences in fur cortisol levels of three migratory bats

**DOI:** 10.1007/s00360-025-01609-0

**Published:** 2025-04-16

**Authors:** Dana M. Green, Christine Gilman, Gabriela Mastromonaco, Erin F. Baerwald, R. Mark Brigham

**Affiliations:** 1 Department of Biology, Laboratory Building LB109, University of Regina, 3737 Wascana Parkway, Regina, SK S4S 0A2 Canada; 2 Reproductive Sciences, Toronto Zoo, 361A Old Finch Avenue, Toronto, ON M1B 5K7 Canada; 3 Ecosystem Science and Management, University of Northern British Columbia, 3333 University Way, Prince George, BC V2N 4Z9 Canada

**Keywords:** Vespertilionidae, Bat, Glucocorticoids, Cortisol, Interspecific, Intraspecific

## Abstract

Understanding how organisms respond to their environments is challenging, especially due to the complex processes related to metabolism, energetics, and reproduction. Glucocorticoids (GCs) are metabolic hormones that provide insight into internal process and may trigger a variety of behaviors. To understand how changes to the environment influence wildlife, we must ideally measure levels of GCs in wildlife populations that are relatively undisturbed by anthropogenic change or natural disaster. In free-ranging mammals, cortisol is a primary GC hormone and can be measured in fur. Bats are the only mammalian order to have evolved true flight and, in North America, most species of bats are in the family Vespertilionidae. By measuring cortisol levels during energetically expensive periods of an animal’s life history, we can document ‘baseline levels’ related to different life stages, sexes, and ages. We collected fur from hoary (*Lasiurus cinereus*), silver-haired (*Lasionycteris noctivagans*), and little-brown Myotis (*Myotis lucifugus*) bats from southern Alberta and Saskatchewan. Herein we report their baseline levels of fur cortisol, noting interspecific differences between species. We found that bats known to migrate longer distances had higher levels compared to bats that migrate shorter distances, and then hibernate. Interestingly, we found no differences in levels between the reproductive stages for any species. Finally, both silver-haired and hoary bats show a strong difference in fur cortisol levels between adults and juveniles. We suggest that the elevated levels are likely associated with events for mothers during lactation which are then incorporated into the pups’ fur while nursing.

**Supplementary Information:**

The online version contains supplementary material available at 10.1007/s00360-025-01609-0.

## Introduction

Understanding how organisms physiologically respond to their environments is challenging, especially due to the complex processes related to metabolism, energetics, and reproduction (Cooke et al. 2014). If researchers want to predict behavioral responses that wildlife may have to changes in the environment, we need to understand aspects of their ecophysiology and ecology. Glucocorticoids, such as biologically active cortisol and inactive cortisone, are metabolic hormones that provide insight into the internal process and potential stressors that organisms experience (reviewed in Landys et al. 2006; Wikelski and Cooke 2006), which may trigger a variety of behaviors.

Across many vertebrate taxa, the secretion and release of GCs is a process involving the activation of the hypothalamic–pituitary–adrenal (HPA) axis (Minton 1994; Gans and Coffman 2021). Normally, GCs are key hormones assisting in regulating energy metabolism, growth, and circadian rhythms (Landys et al. 2006), and are often associated with stimulating migratory behaviors (Jachowski and Singh 2015; Eikenaar 2017; Eikenaar et al. 2018). However, when individuals must cope with environmental changes, GC levels may increase, facilitating shifts in energy balance and thus behavior and physiology (Wingfield and Romero 2015). Prolonged elevation of GCs may cause adverse physiological effects such as reducing reproductive performance (Ching 1983; Suter and Schwartz 1985) and suppressing immune responses (Sapolsky et al. 2000; Tilbrook 2000; Wingfield and Sapolsky 2003).

To understand how changes to the environment act as potential stressors, we can measure levels of GCs in wildlife populations, ideally in populations relatively undisturbed by anthropogenic change or natural disaster. In free-ranging mammals, cortisol is a primary GC hormone and is measured in samples of blood, urine, feces and or fur (Sheriff et al. 2011). Of these, blood, urine, and feces provide an acute view of HPA activity (i.e. within hours, possibly days; Reeder et al. 2004; Constable et al. 2006; Palme 2019), whereas GCs measured in fur reveal longer term levels (i.e. weeks to months); this reflects the circulatory, metabolic and hair growth timelines during which GCs are being deposited in the sample (Macbeth et al. 2010; Mastromonaco et al. 2014; Sandoval-Herrera et al. 2021). Considering the variety of conservation issues facing wildlife, which often involve persistent disturbances to the environment, using hair or fur may provide better insight into longer term effects on populations. Additionally, the collection of fur is minimally invasive, relatively large samples can be collected from each individual and samples are unlikely to be influenced by the stress of capture and handling (Koren et al. 2002; Williams et al. 2009; Macbeth et al. 2010). By measuring cortisol levels during energetically expensive periods of life history, conservation biologists can determine ‘baseline levels’ related to different life stages, sexes, ages, and possibly changes as a result of annual behavioral processes (i.e. migration and hibernation). Bats are the only mammalian order to have evolved true flight, and in North America, most species of migratory bats are in the family Vespertilionidae which is almost exclusively insectivorous (Kunz and Fenton 2003).

Straddling the provincial border of Saskatchewan and Alberta, Canada, the Cypress Hills rise 430 m above the surrounding prairie landscape (Kulig 1996). While migratory routes are largely unknown, beginning in the spring both hoary (*Lasiurus cinereus*) and silver-haired (*Lasionycteris noctivagans*) bats are thought to migrate longer distances (≥ 1000 km) to the region, while little-brown Myotis (*Myotis lucifugus*) bats migrate regionally (< 500 km). All three species arrive in early June to give birth to and raise their young (Willis and Brigham 2005; Willis et al. 2006; Fraser 2011; Bohn 2017). They are locally abundant summer residents and are the predominant species captured along Battle Creek (Green et al. 2020). While all three species migrate into the Cypress Hills, individual hoary and silver-haired bats may pass through *en route* to summering grounds further north (Baerwald et al. 2014), although other reproductive grounds are unknown. Given that the abundance of all three species during the summer reproductive months provided us with an opportunity to measure cortisol levels among different reproductive stages and ages, both between and among species.

Herein, we describe the inter- and intra-specific differences between the baseline levels of three Vespertilionid bats. Bats begin to moult and regrow their fur over a period of weeks in the summer, as early as June (Fraser et al. 2013), during which time GCs are deposited in the growing fur (Stalder and Kirschbaum 2012). Because cortisol is deposited over time in fur, and it is unclear the exact time when bats are actively moulting, changes in cortisol levels may be not evident over time. Additionally, because cortisol levels are species specific (Romero 2004; Crespi et al. 2013; Sandoval-Herrera et al. 2021), we predicted that we would see interspecific variation between bat species. However, because GCs may play a role in both migration and hibernation, a competing hypothesis could be that there would be no differences in fur cortisol levels between species. Glucocorticoids are known to play an important role in modulating reproductive hormones (Wingfield and Sapolsky 2003), and other bat taxa have demonstrated differences in GC levels depending on female reproductive stage and/or sex (Reeder et al. 2004; Sandoval-Herrera et al. 2021). Thus, we also predicted we would find intraspecific differences in GCs depending on reproductive stage and sex for each species.

## Methods

### Study species

Hoary bats (*Lasiurus cinereus*) are long-distance migrants, flying ≥ 1000 km (Fleming and Eby 2003; Weller et al. 2016) between wintering and summering locations. In the summer, after females migrate north, they give birth to pups (normally twins; Shump and Shump 1982), roost solitarily in trees (Willis and Brigham 2005; Klug et al. 2012), and the young of the year become volant and forage on their own at the end of July (Shump and Shump 1982). While it is suspected that hoary bats evolved to migrate as a way to avoid cold northern temperatures and low insect abundance during winter (Cryan et al. 2014), the species is capable of hibernation (Weller et al. 2016; Marín et al. 2021). However, hibernation by this bat has been best studied during migration and during inclement weather after arrival on the summering grounds (Willis et al. 2006).

Similar to hoary bats, silver-haired bats (*Lasionycteris noctivagans)* are classified as “long-distance” migrants, but exhibit ecological similarities and differences compared to hoary and the little-brown Myotis. Silver-haired bats typically have twins and roost in a variety of structures during migration and over the winter (Kunz 1982; Barclay et al. 1988; Perry et al. 2010). Unlike hoary bats, silver-haired bats do not roost solitarily in summer (Barclay et al. 1988; Bohn 2017), and some populations may not be migratory (Nagorsen et al. 1993; Cryan 2003; Fraser et al. 2017). Additionally, there is evidence of winter activity at northern latitudes (Schowalter et al. 1978; Nagorsen et al. 1993; Brigham 1995), similar to more sedentary bats, whereas there is little or no activity by other migratory species at those latitudes (Cryan 2003).

The little-brown Myotis (*Myotis lucifugus)* is considered a regional migrant, having been documented traveling 10–647 km (Fleming and Eby 2003; Norquay et al. 2013). Behaviorally, migratory tree-roosting bats are assumed to have two, long-distance (≥ 1,000 km) annual migrations (spring and fall), whereas regional migrants tend to fly shorter distances from hibernacula to summer roosts (Fleming and Eby 2003; Norquay et al. 2013; Colatskie et al. 2018). Additionally, rather than roosting solitarily, little-brown Myotis tend to roost in large groups in hibernacula during the winter and tree and rock crevices, tree cavities, and/or man-made structures during the summer (Fenton and Barclay 1980). Finally, little-brown Myotis have physiological adaptations to cope with surviving cold winters in situ. The most notable is the ability to hibernate for months (Menaker 1961), which requires individuals acquiring substantial fat reserves prior to entry into seasonal torpor (Kunz et al. 1998; McGuire et al. 2012a).

### Field site

We collected data in the Cypress Hills Interprovincial Park in southwestern Saskatchewan, Canada (49°34′N, 109°53′W) from individuals captured while they foraged along the Battle Creek, the main water body bisecting the park and used as a flyway for all three species (Green et al. 2020). The Cypress Hills are unique in the prairie ecosystem of Saskatchewan because of historical geological processes which left the area as an uplifted, glacial refugium. They are characterized as having 50% grasslands, 45% woodlands, and 5% wetlands (Sauchyn 1993). The wooded areas are a mix of coniferous and deciduous trees where lodgepole pine (*Pinus contorta*), white spruce (*Picea glauca*), and trembling aspen (*Populous tremuloides*) are by far the dominant tree species. In the Cypress Hills, the local abundance of migratory bats has increased based on capture rate data since 2000 (Green et al. 2020). We collected samples during the summer months, from the last week of June through the second week of August in 2019, 2021, and 2022.

### Handling and sample collection

We followed bat handling guidelines published by the American Society of Mammalogists (Sikes 2016). All research conducted was approved by the University of Regina’s President’s Committee on Animal Care and followed permits from Saskatchewan Parks Fish, Wildlife, and Lands branch. To avoid airborne transmission of potentially transmissible disease (such as SARS-CoV-2 virus; COVID-19), we used N95 masks, gloves, and sanitizing procedures while handling bats.

To age bats, we used an external light source to transilluminate the wing of each individual to visualize the cartilaginous zone of the long phalanges. The less mineralized tissue allows more light to pass through and thus appears lighter than bone, indicating an individual was born during the current summer season (i.e. under 1 year of age; Wilkinson and Brunet-Rossinni 2009). We collected standard morphometric measurements (i.e. forearm length, mass, sex, and reproductive status) and if deemed in good health, we also collected fur, fecal, and tissue samples for future use. Using round-tipped dissection scissors, we trimmed 3–10 mg of fur from between the scapula on the dorsal surface, close to the root. The amount of fur collected depended on the species and density of fur, but we ensured collecting 3 mg at a minimum (Sandoval-Herrera et al. 2021). We used tweezers to collect the trimmed fur and placed it in individual paper envelopes. All equipment used was sanitized using 95% ethanol after handling each individual. While there is little documentation on fur moult or growth in bats, our study species are thought to have an annual moult and regrow their fur between June and August (Fraser et al. 2013). We did not directly observe any moulting.

We collected fur samples from a total of 255 bats (113 silver-haired, 70 hoary, and 72 little-brown Myotis) across 3 years (2019, 2021, 2022). Because some samples did not have enough fur, some were not used for analysis. For silver-haired bats, we analyzed samples from 76 adults and 29 juveniles. For hoary bats, we analyzed samples from 34 adults and 28 juveniles. Finally for little-brown Myotis, we analyzed 55 adults and 14 juveniles (Table 1).

**Table 1:** Common and Latin names of each of the study species, and sample sizes within each subset of the data. Subsets with designation “NR” indicate non-reproductive individuals of both sexes and were used for models investigating the interspecific differences between species and the intraspecific differences between ages of adults and juveniles

Species		*n*							
Common	Latin	NR Adult		NR Juvenile		Reproductive condition (Adult ♀)			
		♀	♂	♀	♂	N	P	L	PL
Silver-haired Bat	*Lasionycteris noctivagans*	8	23	20	9	8	6	26	13
Hoary Bat	*Lasiurus cinereus*	2	11	13	15	2	1	16	4
Little-brown Myotis	*Myotis lucifugus*	9	21	10	4	9	4	16	5

### Extraction and quantification of cortisol

All chemicals used in the process to extract GCs were obtained from Sigma-Aldrich Canada Co., Oakville, ON, Canada, unless otherwise stated. Fur samples were analyzed for GC concentrations at the Toronto Zoo Endocrinology Lab (Toronto, ON, Canada) following the methods of Sandoval-Herrera et al. (2021). Hormones were extracted from fur samples as previously described (Sandoval-Herrera et al. 2021) within 3–23 months (83–674 days) since collection. Fur extracts were reconstituted at a threefold concentration (420 µl sample reconstituted in 140 µl buffer). One sample (silver-haired) with high hormone concentration was re-assayed at a 1:4 dilution. Samples with lower concentrations (hoary, n = 2 and little-brown Myotis, n = 6) were re-assayed at tenfold concentration. Additionally, three samples (little-brown Myotis) had insufficient fur to be re-assayed and were assigned a cut-off value based on the lower limit of quantitation for the assay (< 10 ng/g), Cortisol concentrations were determined using an enzyme immunoassay (EIA) previously described (Dulude-de Broin et al. 2019) with antibody (R4866, C. Munro, University of California, Davis) and horseradish peroxidase dilutions adjusted 1:10,200 and 1:33,400, respectively as done for Sandoval-Herrera et al. (2021). Samples were assayed in duplicate except for one silver-haired and nine little-brown Myotis samples with insufficient fur were assayed with one replicate, which were not included within the analysis. Results are presented as nanograms of cortisol per gram of fur.

### Enzyme immunoassay validation

Parallel displacement between the standard curve and serial dilutions of fur extract for each species was used as an indirect measure of assay suitability and dose–response relationship. Pools were made from 11–15 fur extracts, evaporated and reconstituted in assay buffer (little-brown Myotis: 32-fold concentration, hoary and silver-haired: eightfold concentration) then serially diluted two-fold in assay buffer and compared to the respective standard curve. The data were plotted as log (relative dose) vs. percent antibody bound using Microsoft Excel. Linear regression analysis determined the slopes of the lines within the linear portion of the curves and each pair of curves were compared (Soper 2025) where *p* > 0.05 indicates the slopes are not significantly different and thus parallel. Samples were assayed at the dilution factor that corresponded to 50% binding of the serially diluted pool for each species. Serial dilutions of pooled fur extract showed parallel displacement with the cortisol standard curve for all species tested (hoary: t = 1.66, *p* = 0.16, df = 5; silver-haired: t = 2.18, *p* = 0.08, df = 5; little-brown Myotis: t = 0.03*, p* = 0.98, df = 6; supplementary materials). Inter-assay coefficients of variation at 24% binding were 8.6%, 5.5% and 13.7%, and at 57% binding were 7.5%, 13.1% and 13.6% for hoary, silver-haired and little-brown Myotis respectively. The intra-assay CV was 8.5%.

### Data analysis

To explore differences in baseline levels of fur cortisol concentration between and within species, we used R-Studio (Version 1.4.1106) to run generalized linear models (GLMs) with a log-link gamma distribution to account for non-normality, and analysis of variance models using the ‘aov’ function in R (R core Team 2021). To explore general differences between the three species of bats, we separated the full dataset into “adults” and “juveniles”, with general baseline levels being reflective of non-reproductive adults, and ran separate models for each age group. While the initial models explore mean differences between species, we note that cortisol concentrations are species specific and thus results should be interpreted with care and used descriptively.

To first establish a baseline level for each species, we subset the data to only include non-reproductive, adult individuals. However, because of smaller sample sizes of non-reproductive individuals within each species, we kept the sexes grouped together. To see if body mass and fur cortisol concentration have a relationship, we ran models for each species separating between adults and juveniles. To compare how fur cortisol concentrations differed between age classes for each species and to exclude possible effects of reproductive state, we used the subset data of non-reproductive individuals of both sexes and ran our model including age (JV/Adult), year, and month. Finally, to understand if reproductive status of females influenced cortisol concentration, we ran our model with the data subset based on sex and age (adult females only) for each species and included year, month, reproductive status, and an interaction between month and reproductive status. We used Tukey’s post hoc tests for significant covariates and compared effect sizes based on the degree of overlap using 95% confidence intervals.

## Results

### Interspecific differences

**Fig. 1:**
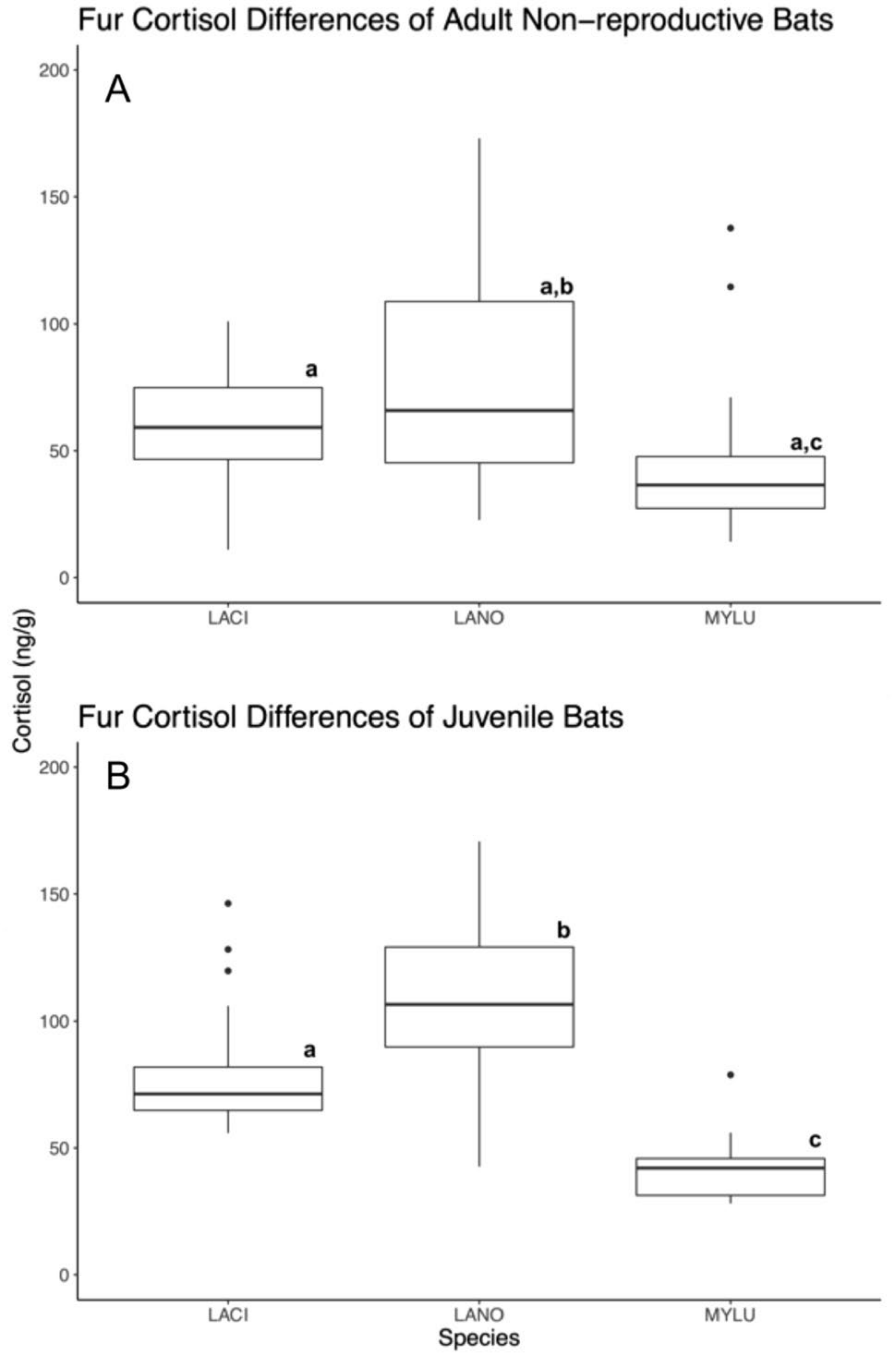
Interspecific differences of fur cortisol concentrations between hoary (*L. cinereus;* LACI), silver-haired (*Ln. noctivagans;* LANO), and little-brown Myotis (*M. lucifugus;* MYLU) bats. The top boxplot (**A**) represents the mean fur cortisol concentrations for non-reproductive adults and the bottom (**B**) boxplot represents fur cortisol concentrations for juveniles. The length of the boxes indicates the interquartile range, length of whickers indicating to total minimum and maximum of each species group, and outlier points within the dataset that exceed the quartile range. Letters above the boxes indicates whether groups differed significantly from each other

Mean fur cortisol concentrations of adult non-reproductive bats were significantly different, whereas long-distance migratory species (silver-haired and hoary bats) exhibit higher fur cortisol concentrations when compared to little-brown Myotis bats (F_2, 71_ = 7.38; *p* < 0.01; Fig. 1). Silver-haired and hoary bats did not differ significantly from each other (*p* > 0.1), nor did hoary bats when compared to little-brown Myotis bats (*p* > 0.1). However, silver-haired bats exhibited significantly higher fur cortisol concentration compared to little-brown Myotis bats (*p* < 0.01; Fig. 1). We found no effect of year or month for the levels in non-reproductive individuals.

Mean fur cortisol concentrations of juvenile bats were significantly different, whereas long-distance migratory species (silver-haired and hoary bats) exhibit higher fur cortisol concentrations when compared to little-brown Myotis bats (F_2, 68_ = 33.91; *p* < 0.01; Fig. 1). Juvenile silver-haired exhibited the highest cortisol concentration for the species, differing significantly from both hoary (*p* < 0.01) and little brown Myotis (*p* < 0.01) bats. We found no effect of year or month for juveniles (*p* > 0.05).

### Intraspecific differences

**Fig. 2:**
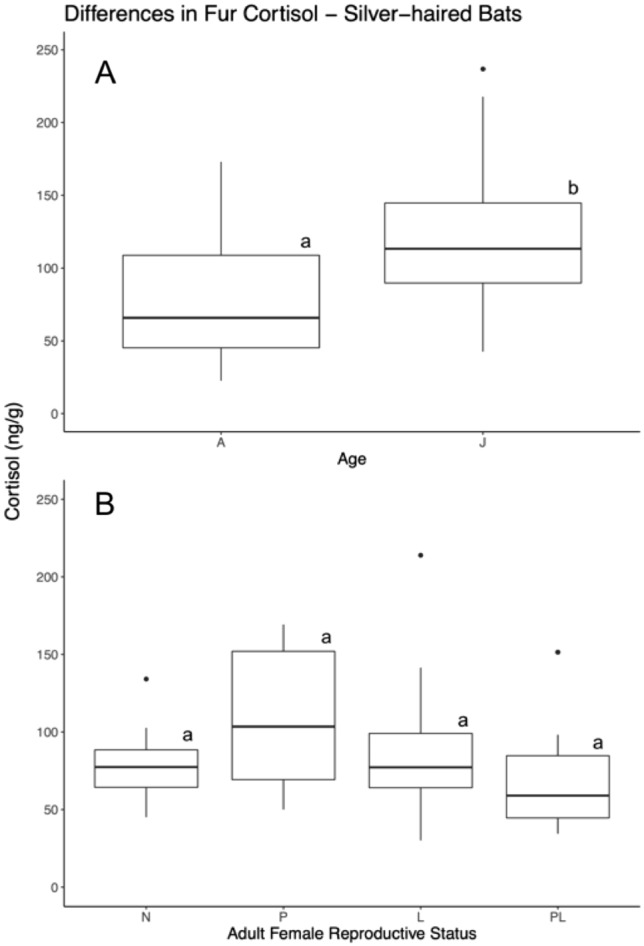
Intraspecific differences in fur cortisol concentrations for silver-haired bats (*Ln. noctivagans*). The top boxplot (**A**) depicts differences in fur cortisol concentrations between non-reproductive adults and juveniles, and the bottom boxplot (**B**) represents differences in fur cortisol concentrations based on the reproductive stages of adult females. Boxes in ‘B’ depict differences between non-reproductive (N), pregnant (P), lactating (L), post-lactating (PL), and scrotal (S) individuals. The length of the boxes indicates the interquartile range, length of whickers indicating to total minimum and maximum of each species group, and outlier points within the dataset that exceed the quartile range. Letters above the boxes indicates whether groups differed significantly from each other

Mean baseline fur cortisol concentrations for adult non-reproductive silver-haired bats were 74.97 ng/g ± 38.69 SD. Juvenile silver-haired bats exhibited a higher mean fur cortisol concentration of 121.32 ng/g ± 44.24 SD. Our model comparing non-reproductive adult and juvenile silver-haired bats found that juveniles exhibit significantly higher fur cortisol concentrations (F_1,58_ = 20.54; *p* < 0.01; Table 2). Cortisol concentration in juvenile silver-haired bats had a mean difference of 46.35 ng/g higher relative to adults (Fig. 2). Additionally, we found that ‘month’ predicted cortisol concentration which increased from July to August (F_1,58_ = 6.62; *p* = 0.01). Our model comparing all of the different reproductive stages of adult female silver-haired bats found no effect of reproductive condition (F_3,49_ = 1.81; *p* > 0.1), month, the interaction of month and condition, nor year. Sample sizes for pregnant females were low (n = 6), so these results need to be interpreted conservatively.

**Table 2:** Analysis of variance results based on a generalized linear model with a log-link gamma distribution, predicting fur cortisol concentration based on species demographics for silver-haired (*Ln. noctivagans*), hoary (*L. cinereus*), and little-brown Myotis (*M. lucifugus*) bats. Above the dashed line, for each species, indicates the model results that included non-reproductive individuals of each species, both adults and juveniles, along with predictive variables and interactions of variables. Below the dashed line indicates the adult-female only models and its predictive variables

Species		Variable	*df*	F	*p*-value
Silver-haired Bat	*Lasionycteris noctivagans*	Mass	1	1.11	> 0.05
		Sex	1	0.43	> 0.05
		Age	1	20.19	< 0.01*
		Month	1	6.51	0.01*
		Year	2	0.35	> 0.05
		Mass*Month	1	1.84	> 0.05
		Mass*Age	1	0.94	> 0.05
		Repro. status	3	1.81	> 0.05
		Month	2	0.60	> 0.05
		Year	2	2.54	> 0.05
		Repro*Month	2	1.80	> 0.05
Hoary Bat	*Lasiurus cinereus*	Mass	1	4.44	< 0.05*
		Sex	1	0.86	> 0.05
		Age	1	7.45	0.01*
		Month	2	2.02	> 0.05
		Year	2	2.46	> 0.05
		Mass*Month	1	0.74	> 0.05
		Mass*Age	1	0.45	> 0.05
		Repro. status	3	0.13	> 0.05
		Month	2	8.36	< 0.01*
		Year	2	2.51	> 0.05
		Repro*Month	1	0.62	> 0.05
Little-brown Myotis	*Myotis lucifugus*	Mass	1	0.35	> 0.05
		Sex	1	2.86	> 0.05
		Age	1	0.07	> 0.05
		Month	2	0.55	> 0.05
		Year	1	0.58	> 0.05
		Mass*Month	1	0.77	> 0.05
		Mass*Age	1	0.26	> 0.05
		Repro. status	3	1.22	> 0.05
		Month	2	2.67	> 0.05
		Year	1	0.21	> 0.05
		Repro*Month	1	0.10	> 0.05

*Designates alpha < 0.05 and statistical significance

**Fig. 3:**
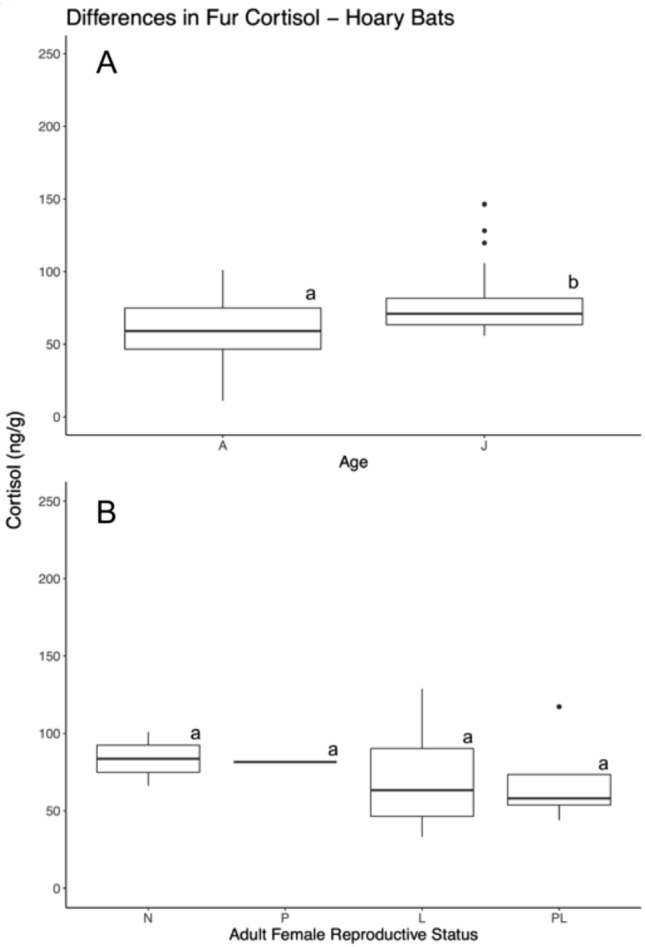
Intraspecific differences in fur cortisol concentrations for hoary bats (*L. cinereus*). The top boxplot (**A**) depicts differences in fur cortisol concentrations between non-reproductive adults and juveniles, and the bottom boxplot (**B**) represents differences in fur cortisol concentrations based on the reproductive stages of adult females. Boxes in ‘B’ depict differences between non-reproductive (N), pregnant (P), lactating (L), post-lactating (PL), and scrotal (S) individuals. The length of the boxes indicates the interquartile range, length of whickers indicating to total minimum and maximum of each species group, and outlier points within the dataset that exceed the quartile range. Letters above the boxes indicates whether groups differed significantly from each other

Mean baseline fur cortisol concentrations for adult non-reproductive hoary bats were 57.51 ng/g ± 27.19. Juvenile hoary bats exhibited a higher mean fur cortisol concentration of 77.75 ng/g ± 22.22. For this species, cortisol concentration within the full model was predicted by mass (F_1,39_ = 4.44; *p* < 0.05; Table 2) and age (F_1,39_ = 7.45; *p* = 0.01; Table 2, Fig. 3). Juvenile hoary bats had a mean difference of 20.24 ng/g higher relative to adults (Fig. 3). Our model comparing the different reproductive stages of adult female hoary bats found no effect of reproductive condition (F_3,21_ = 0.13; *p* > 0.1; Fig. 3), interaction of month and condition, nor year. There was a significant effect of month alone (F_3,21_ = 8.36; *p* < 0.01) reflecting the higher cortisol concentration in June. However, because of the low sample size for pregnant hoary bats (n = 1) and bats that were captured in June (n = 3), our results likely do not capture the extent of differences between groups.

**Fig. 4:**
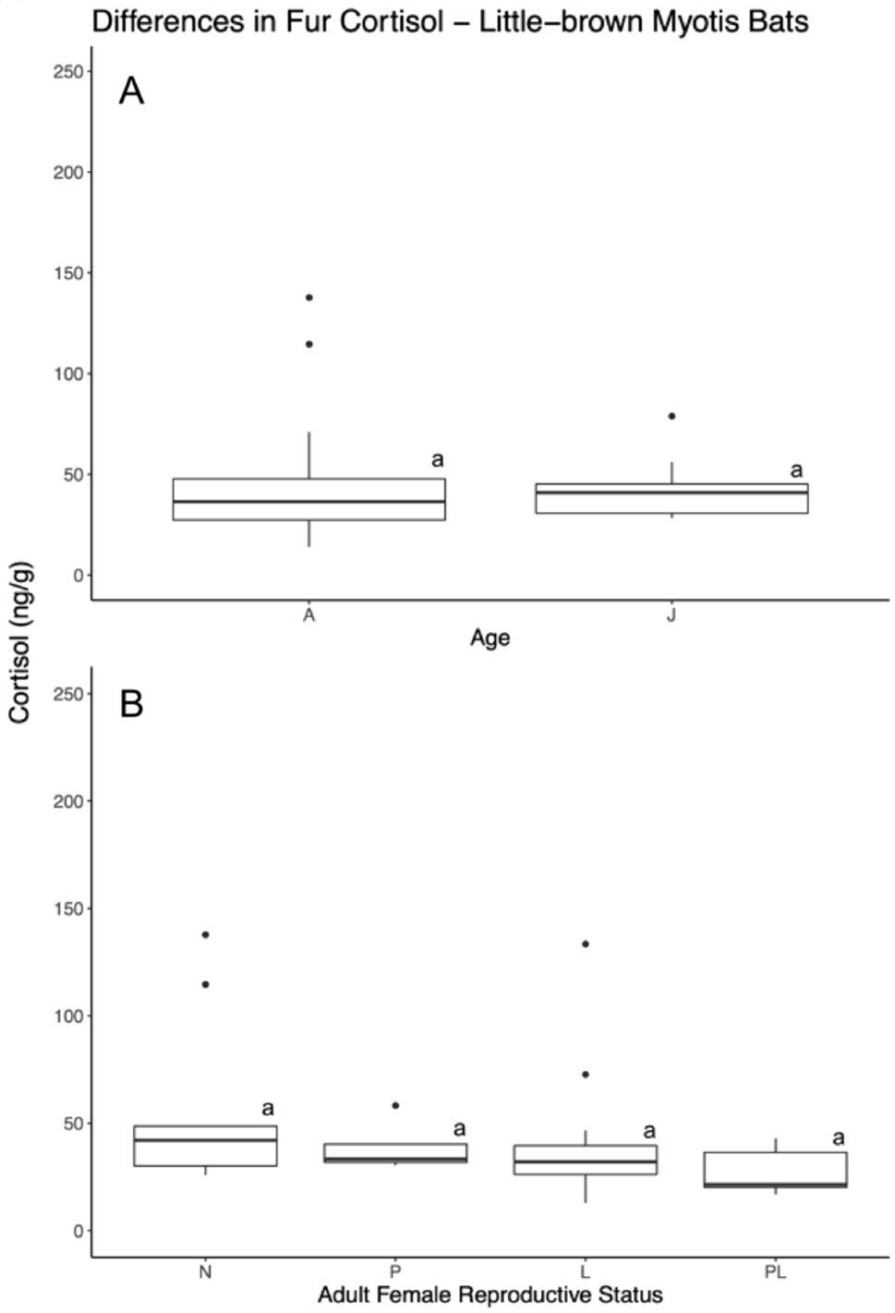
Intraspecific differences in fur cortisol concentrations for little-brown Myotis bats (*M. lucifugus*). The top boxplot (**A**) depicts differences in fur cortisol concentrations between non-reproductive adults and juveniles, and the bottom boxplot (**B**) represents differences in fur cortisol concentrations based on the reproductive stages of adult females. Boxes in ‘B’ depict differences between non-reproductive (N), pregnant (P), lactating (L), post-lactating (PL), and scrotal (S) individuals. The length of the boxes indicates the interquartile range, length of whickers indicating to total minimum and maximum of each species group, and outlier points within the dataset that exceed the quartile range. Letters above the boxes indicates whether groups differed significantly from each other

Mean baseline fur cortisol concentrations for adult non-reproductive little-brown Myotis bats were 43.31 ng/g ± 26.91. Juvenile little-brown Myotis bats have a mean fur cortisol concentration of 41.23 ng/g ± 13.61. For little-brown Myotis bats there were no differences between non-reproductive adults and juveniles, nor was cortisol concentration predicted by month or year (Table 2, Fig. 4). Our model comparing all of the different reproductive stages of adult female little-brown Myotis bats found no effect of reproductive condition (F_3,31_ = 1.22; *p* > 0.1, Fig. 4), month, interaction of month and condition, nor year. However, there was also a small sample size of pregnant (n = 4) and post-lactating individuals (n = 5) so these results should be interpreted conservatively.

## Discussion

For mammals, cortisol extracted from fur can give conservation biologists a glimpse into how individuals physiologically react to long term environmental changes and possible stressors, but this requires knowledge of baseline levels for comparisons. Additionally, understanding when a species moults and regrows fur is important because the cortisol profile represents the time of fur growth. While there is not much literature on fur growth in bats, many temperate bat species are thought to have an annual moult cycle during the summer months (Fraser et al. 2013). We collected fur samples from three species of different genera within the same family of North American bats during the summer, and report ‘baseline’ levels of cortisol concentration based on non-reproductive adults. Compared to the results of Sandoval-Herrera et al. (2021), we found lower concentrations of fur cortisol levels in all 3 species compared to fur cortisol levels in 5 families encompassing 18 species of tropical bats, including species in the same genera as two of our species (*Lasiurus* and *Myotis*). The southern yellow bat (*Lasiurus ega)* is documented to have a mean fur cortisol concentration of 142.83 ng/g ± 18.14, whereas we report a mean of 57.51 ng/g ± 27.19 for adult non-reproductive hoary bats (*Lasiurus cinereus*), and the cave Myotis (*Myotis velifer)* is reported to have a mean cortisol concentration of 211.65 ng/g ± 170.79 whereas we report for the little-brown Myotis a mean of 43.31 ng/g (Sandoval-Herrera et al. 2021). Because tropical and temperate species occupy notably different habitats and exhibit different seasonal behaviors, it is possible species exhibit both intrinsic and extrinsic differences, resulting in different long-term cortisol concentrations. Considering that there are dramatic differences in cortisol concentrations across bat taxa, further investigation comparing the values derived from fur, feces, and plasma are warranted (Koren et al. 2019).

### Interspecific differences

In agreement with existing literature, we found species specific differences in baseline levels of GCs, though hoary and silver-haired bats were similar (Fig. 1; Romero 2004; Crespi et al. 2013; Sandoval-Herrera et al. 2021). We suggest there may be competing reasons for this trend; first, it is possible that migratory behavior is partially regulated by the release of GCs, thus temperate migratory bats may have higher cortisol levels comparatively (reviewed in birds, Bauer and Watts 2021; Eikenaar 2017; Eikenaar et al. 2018). While long-distance migratory animals often occupy more seasonal environments and take advantage of seasonal resources (Thorup et al. 2017) or stopovers during migration (Hedenström 2008; McGuire et al. 2012b; Jonasson and Guglielmo 2019), not all exhibit elevated GC concentrations in preparation for migration. However, some migratory mammals such as mule deer (*Odocoileus hemionus*) exhibit acute elevations in fecal GC metabolites during migration while at stopover sites (Jachowski et al. 2018), thus it may be that migratory bats exhibit hormonal shifts to prepare for fall migration. To get a better understanding of how GCs change during migration, we suggest sampling a variety of biomarker substrates, including fur, feces, and plasma, strategically throughout the year to capture the migratory and non-migratory windows.

Along with influencing migration behaviors, cortisol also plays a role in regulating hibernation behavior. For example in another long-term hibernator (brown bears; > 5 months), there are significantly heightened levels of serum cortisol *during* hibernation compared to the pre-hibernation hyperphagia period (Vella et al. 2020). Further, it has been demonstrated that cortisol in bears plays a vital role in preventing hypoglycemia via reduction of glucose uptake in tissues and the stimulation of lipolysis, both physiological functions needed for the metabolic demands of hibernation (Djurhuus et al. 2004; Kuo et al. 2015; Vella et al. 2020). While hibernation is documented in each of our study species (Marín et al. 2021; De Freitas 2023), little-brown Myotis bats hibernate consistently over a longer period of time compared to the other two species. Given that cortisol may play a role in regulating hibernation behaviour, but that we sampled little-brown Myotis before the hibernation season (approximately October–April), we may not have captured the time frame where their cortisol concentrations could be higher. Little-brown Myotis are noted to exhibit increased plasma cortisol during hibernation (Gustafson and Belt 1981), thus it may be useful to sample fur during hibernation to explore whether there are differences between the summer and winter months. Given that all 3 species of bats we studied exhibit different migration and hibernation behaviors, and we demonstrate that the long-distance migrants both have elevated cortisol comparatively, bats may be good model species for understanding how cortisol is secreted depending on behavior and life history strategy. It has been shown that fecundity is a likely predictor of bat fur cortisol concentration (Sandoval-Herrera et al. 2021). Both silver-haired and hoary bats likely give birth to twins in our study area, while little-brown Myotis have a single pup. It is possible that the difference in fecundity may explain the differences in cortisol concentrations, documented and predicted in tropical species. However, colony size, roost durability, and basal metabolic rate did not predict levels (Sandoval-Herrera et al. 2021).

### Intraspecific differences

The only species to show an effect of month are hoary bats, having a positive relationship as the summer progressed from July to August for both adults and juveniles. However, given our sample size for hoary bats in June was low, our results may not be reflective of the local population and should be interpreted with caution. Both silver-haired and hoary bats exhibited intraspecific differences, particularly between adults and juveniles. Based on the means, juvenile silver-haired bats had fur cortisol concentrations 62% higher than adults. Juveniles of other mammal species such as black-footed ferrets (*Mustela nigripes*; Santymire et al. 2021), Egyptian mongoose (*Herpestes ichneumon*; Azevedo et al. 2019), and mountain goats (*Oreamnos americanus*; Dulude-de Broin et al. 2019) have been reported to have higher concentrations of GCs compared to adults, attributed to growing their fur during times of rapid overall growth and metabolic demand. However, we suggest it may be likely an endogenous effect. Considering we found no effect of year, it suggests that higher cortisol concentrations in juveniles are consistent. Although not as pronounced as with silver-haired bats, we found a similar trend within hoary bats whereas juveniles have elevated cortisol levels compared to non-reproductive adults by approximately 35%. Additionally, because the juvenile bats would have grown their fur completely while nursing and have protection from the environment within the roost, we suggest it is unlikely that their environmental experiences are the cause of increased cortisol levels. It is plausible that these concentrations are derived from cortisol ingested with mother's milk, as seen in other species where maternal stress hormones are transferred to the offspring through lactation (Catalani et al. 2000; Sheriff et al. 2010; Dantzer et al. 2013; Stead et al. 2022). Because we found the same trend in both of our “long-distance” migratory bats, we further suggest that HPA activity plays a role in migration behavior and physiology. As seen with corticosterone in birds (reviewed by Bauer and Watts 2021), HPA activity in bats may influence departure time for migration and assist with sustaining longer flights.

Interestingly and counter to our predictions, we did not observe any differences between reproductive stages for any of our bat species. In contrast to previous work on little-brown Myotis that noted elevated plasma cortisol in late-stage pregnancy (Reeder et al. 2004), we did not record any differences in pregnant females within our models. However, other studies that sampled bat fur and not plasma also found no differences of reproductive stage (Sandoval-Herrera et al. 2021). However, given that we had small sample sizes, especially for pregnant females, we may not have captured the true values for the demographic. Additonally, because pregnancy and lacatation are shorter-term physiological phenomina, it may be more informative to use plasma or feces to better understand the influence of GCs on reproductive stages. Among mammals, temperate bats have a unique reproducitve cycle whereby breeding occurs in the fall, followed by a delayed pregnancy until spring (Fenton and Barclay 1980; Kunz 1982; Shump and Shump 1982). Thus, different bat species may exhibit unique cortisol profiles in regards to reproductive cycles. Other predictors, such as sex differences, has been noted in many mammalian species (Hau et al. 2016). Though limited, literature on free-ranging mammals suggests differences between sexes is common, with females often having more elevated baseline concentrations (Boonstra et al. 2001; Reeder and Kramer 2005; Bechshøft et al. 2011). Similar to Sandoval-Herrera et al. (2021) that found 14 of the 18 species exhibited no differences in fur cortisol concentrations between sexes, we also found that sex did not predict cortisol concentration.

## Summary

Herein we describe the basal levels of fur cortisol concentrations for three species of North American Vespertilionid bat. We saw interspecific differences between species, whereas bats known to migrate longer distances (> 1000 km) had higher levels compared to a bat that migrates shorter distances (< 500 km), and then hibernates. We thus suggest that there are differences in HPA activity depending on migration strategy for bat species. Interestingly, we found no differences in reproductive stage for any of our species. While this agrees with literature on other fur cortisol concentration derived from bat species (Sandoval-Herrera et al. 2021), it also is inconsistent with other studies of little-brown Myotis (Reeder et al. 2004). Finally, we found that for juvenile, long-distance migratory species, they exhibit higher cortisol concentrations compared to adults. We suggest that there may be a link between maternal cortisol secretion from milk that may play a role in early development for offspring.

## Supplementary Information

Below is the link to the electronic supplementary material.

Supplementary file1 (DOCX 29 KB)

## Data Availability

The data that support the findings of this study are available from the corresponding author, Dana M. Green (dana.green.eco@gmail.com), upon reasonable request.

## References

[CR1] Azevedo A, Bailey L, Bandeira V et al (2019) Age, sex and storage time influence hair cortisol levels in a wild mammal population. PLoS ONE 14:e0221124. 10.1371/journal.pone.0221124 31398221 10.1371/journal.pone.0221124 PMC6688795

[CR2] Baerwald EFB, Atterson WPP, Barclay RMR (2014) Origins and migratory patterns of bats killed by wind turbines in southern Alberta: evidence from stable isotopes. Ecosphere 5:1–17. 10.1890/ES13-00380.1

[CR3] Barclay RMR, Faure PA, Farr DR (1988) Roosting behavior and roost selection by migrating silver-haired bats (*Lasionycteris noctivagans*). J Mammal 69:821–825

[CR4] Bauer CM, Watts HE (2021) Corticosterone’s roles in avian migration: Assessment of three hypotheses. Horm Behav 135:105033. 10.1016/j.yhbeh.2021.105033 34273707 10.1016/j.yhbeh.2021.105033

[CR5] Bechshøft TØ, Sonne C, Dietz R et al (2011) Cortisol levels in hair of East Greenland polar bears. Sci Total Environ 409:831–834. 10.1016/j.scitotenv.2010.10.047 21144554 10.1016/j.scitotenv.2010.10.047 PMC3019279

[CR6] Bohn SJ (2017) Tall timber: Roost tree selection of reproductive female silver-haired bats (*Lasionycteris noctivagans*). Masters Thesis, University of Regina

[CR7] Boonstra R, McColl CJ, Karels TJ (2001) Reproduction at all costs: the adaptive stress response of male arctic ground squirrels. Ecology 82:1930–1946. 10.1890/0012-9658(2001)082[1930:RAACTA]2.0.CO;2

[CR8] Brigham RM (1995) A winter record for the silver-haired bat in Saskatchewan. Blue Jay 53:168

[CR9] Catalani A, Casolini P, Scaccianoce S et al (2000) Maternal corticosterone during lactation permanently affects brain corticosteroid receptors, stress response and behaviour in rat progeny. Neuroscience 100:319–325. 10.1016/S0306-4522(00)00277-3 11008169 10.1016/s0306-4522(00)00277-3

[CR10] Ching M (1983) Morphine suppresses the proestrous surge of GnRH in pituitary portal plasma of rats. Endocrinology 112:2209–2211. 10.1210/endo-112-6-2209 6406211 10.1210/endo-112-6-2209

[CR11] Colatskie SN, Layne JT, Gerdes C, Robbins LW (2018) Recent migratory movements of gray bats (Myotis grisescens) in Missouri: potential to spread Pseudogymnoascus destructans? Bat Research News 49:11–19

[CR12] Constable S, Parslow A, Dutton G et al (2006) Urinary cortisol sampling: a non-invasive technique for examining cortisol concentrations in the Weddell seal, *Leptonychotes weddellii*. Zoo Biol 25:137–144. 10.1002/zoo.20088

[CR13] Cooke SJ, Blumstein DT, Buchholz R et al (2014) Physiology, behavior, and conservation. Physiol Biochem Zool 87:1–14. 10.1086/671165 24457917 10.1086/671165

[CR14] Crespi EJ, Williams TD, Jessop TS, Delehanty B (2013) Life history and the ecology of stress: how do glucocorticoid hormones influence life-history variation in animals? Funct Ecol 27:93–106. 10.1111/1365-2435.12009

[CR15] Cryan PM (2003) Seasonal distribution of migratory tree bats America. J Mammal 84:579–593

[CR16] Cryan PM, Stricker CA, Wunder MB (2014) Continental-scale, seasonal movements of a heterothermic migratory tree bat. Ecol Appl 24:602–616 24988763 10.1890/13-0752.1

[CR17] Dantzer B, Newman AEM, Boonstra R et al (2013) Density triggers maternal hormones that increase adaptive offspring growth in a wild mammal. Science 340:1215–1217. 10.1126/science.1235765 23599265 10.1126/science.1235765

[CR18] De Freitas E (2023) Winter roosting ecology of silver-haired bats (Lasionycteris noctivagans) in southern British Columbia. University of Northern British Columbia, Master of Science

[CR19] Djurhuus CB, Gravholt CH, Nielsen S et al (2004) Additive effects of cortisol and growth hormone on regional and systemic lipolysis in humans. Am J Physiol Endocrinol Metab 286:E488–E494. 10.1152/ajpendo.00199.2003 14600073 10.1152/ajpendo.00199.2003

[CR20] Dulude-de Broin F, Côté SD, Whiteside DP, Mastromonaco GF (2019) Faecal metabolites and hair cortisol as biological markers of HPA-axis activity in the Rocky mountain goat. Gen Comp Endocrinol 280:147–157. 10.1016/j.ygcen.2019.04.022 31009603 10.1016/j.ygcen.2019.04.022

[CR21] Eikenaar C (2017) Endocrine regulation of fueling by hyperphagia in migratory birds. J Comp Physiol A Neuroethol Sens Neural Behav Physiol 203:439–445. 10.1007/s00359-017-1152-1 28213760 10.1007/s00359-017-1152-1

[CR22] Eikenaar C, Müller F, Rüppel G, Stöwe M (2018) Endocrine regulation of migratory departure from stopover: Evidence from a longitudinal migratory restlessness study on northern wheatears. Horm Behav 99:9–13. 10.1016/j.yhbeh.2018.01.008 29408015 10.1016/j.yhbeh.2018.01.008

[CR23] Fenton MB, Barclay RMR (1980) Myotis lucifugus. Mamm Species 142:1–8

[CR24] Fleming TH, Eby P (2003) Ecology of bat migration. In: Kunz TH, Fenton MB (eds) Bat ecology. University of Chicago Press, Chicago, Illinois, pp 156–208

[CR25] Fraser EE, Longstaffe FJ, Fenton MB (2013) Moulting matters: The importance of understanding moulting cycles in bats when using fur for endogenous marker analysis. Can J Zool 91:533–544. 10.1139/cjz-2013-0072

[CR26] Fraser EE, Brooks D, Longstaffe FJ (2017) Stable isotope investigation of the migratory behavior of silver-haired bats (Lasionycteris noctivagans) in eastern North America. J Mammal 98:1225–1235. 10.1093/jmammal/gyx085

[CR27] Fraser EE (2011) Stable isotope analyses of bat fur: Applications for investigating North American bat migration. Doctor of Philosophy. The University of Western Ontario

[CR28] Gans IM, Coffman JA (2021) Glucocorticoid-mediated developmental programming of vertebrate stress responsivity. Front Physiol 12:812195. 10.3389/fphys.2021.812195 34992551 10.3389/fphys.2021.812195 PMC8724051

[CR29] Green DM, McGuire LP, Vanderwel MC et al (2020) Local trends in abundance of migratory bats across 20 years. J Mammal 101:1542–1547. 10.1093/jmammal/gyaa154

[CR30] Gustafson A, Belt W (1981) The adrenal cortex during activity and hibernation in the male little brown bat, Myotis lucifugus: Annual rhythm of plasma cortisol levels. Gen Comp Endocrinol 44:269–278 7286611 10.1016/0016-6480(81)90001-0

[CR31] Hau M, Casagrande S, Ouyang JQ, Baugh AT (2016) Glucocorticoid-mediated phenotypes in vertebrates. In: Naguib M, Mitani JC, Simmons LW, Barrett L, Healy S, Zuk M (eds) Advances in the study of behavior. Elsevier, pp 41–115. 10.1016/bs.asb.2016.01.002

[CR32] Hedenström A (2008) Adaptations to migration in birds: Behavioural strategies, morphology and scaling effects. Phil. Trans. R. Soc. B 363:287–299. 10.1098/rstb.2007.2140 17638691 10.1098/rstb.2007.2140 PMC2606751

[CR33] Jachowski DS, Singh NJ (2015) Toward a mechanistic understanding of animal migration: incorporating physiological measurements in the study of animal movement. Conserv Physiol 3:cov035. 10.1093/conphys/cov035 10.1093/conphys/cov035 PMC4778435 27293720

[CR34] Jachowski DS, Kauffman MJ, Jesmer BR, et al (2018) Integrating physiological stress into the movement ecology of migratory ungulates: a spatial analysis with mule deer. Conserv Physiol 6: coy054. 10.1093/conphys/coy054 10.1093/conphys/coy054 PMC6161405 30279991

[CR35] Jonasson KA, Guglielmo CG (2019) Evidence for spring stopover refuelling in migrating silver-haired bats (Lasionycteris noctivagans). Can J Zool 97:961–970. 10.1139/cjz-2019-0036

[CR36] Klug BJ, Goldsmith DA, Barclay RMR (2012) Roost selection by the solitary, foliage-roosting hoary bat (*Lasiurus cinereus*) during lactation. Can J Zool 90:329–336. 10.1139/z11-139

[CR37] Koren L, Mokady O, Karaskov T et al (2002) A novel method using hair for determining hormonal levels in wildlife. Anim Behav 63:403–406. 10.1006/anbe.2001.1907

[CR38] Koren L, Bryan H, Matas D et al (2019) Towards the validation of endogenous steroid testing in wildlife hair. J Appl Ecol 56:547–561. 10.1111/1365-2664.13306

[CR39] Kulig JJ (1996) The glaciation of the Cypress Hills of Alberta and Saskatchewan and its regional implications. Quatern Int 32:53–77

[CR40] Kunz TH (1982) Lasionycteris noctivagans. Mamm Species 172:1–5

[CR41] Kunz TH, Fenton MB (2003) Bat Ecology. University of Chicago Press, Chicago, Illinois

[CR42] Kunz TH, Wrazen JA, Burnett CD (1998) Changes in body mass and fat reserves in pre-hibernating little brown bats (Myotis lucifugus). Ecoscience 5:8–17. 10.1080/11956860.1998.11682443

[CR43] Kuo T, McQueen A, Chen T-C, Wang J-C (2015) Regulation of glucose homeostasis by glucocorticoids. In: Wang J-C, Harris C (eds) Glucocorticoid signaling. Springer, New York, New York, NY, pp 99–126 10.1007/978-1-4939-2895-8_5 PMC6185996 26215992

[CR44] Landys MM, Ramenofsky M, Wingfield JC (2006) Actions of glucocorticoids at a seasonal baseline as compared to stress-related levels in the regulation of periodic life processes. Gen Comp Endocrinol 148:132–149. 10.1016/j.ygcen.2006.02.013 16624311 10.1016/j.ygcen.2006.02.013

[CR45] Macbeth BJ, Cattet MRL, Stenhouse GB et al (2010) Hair cortisol concentration as a noninvasive measure of long-term stress in free-ranging grizzly bears (Ursus arctos): considerations with implications for other wildlife. Can J Zool 88:935–949. 10.1139/Z10-057

[CR46] Marín G, Ramos-H D, Cafaggi D et al (2021) Challenging hibernation limits of hoary bats: the southernmost record of Lasiurus cinereus hibernating in North America. Mamm Biol 101:287–291. 10.1007/s42991-020-00080-4

[CR47] Mastromonaco GF, Gunn K, McCurdy-Adams H, et al (2014) Validation and use of hair cortisol as a measure of chronic stress in eastern chipmunks (Tamias striatus). Conservation Physiology 2:cou055. 10.1093/conphys/cou055 10.1093/conphys/cou055 PMC4732495 27293676

[CR48] McGuire LP, Fenton MB, Guglielmo CG (2012a) Phenotypic flexibility in migrating bats: seasonal variation in body composition, organ sizes and fatty acid profiles. J Exp Biol 216:800–808. 10.1242/jeb.072868 10.1242/jeb.072868 23408801

[CR49] McGuire LP, Guglielmo CG, Mackenzie SA, Taylor PD (2012b) Migratory stopover in the long-distance migrant silver-haired bat, *Lasionycteris noctivagans*. J Anim Ecol 81:377–385. 10.1111/j.1365-2656.2011.01912.x 21954938 10.1111/j.1365-2656.2011.01912.x

[CR50] Menaker M (1961) The free running period of the bat clock; seasonal variations at low body temperature. J Cell Comp Physiol 57:81–86 13769330 10.1002/jcp.1030570204

[CR51] Minton JE (1994) Function of the hypothalamic-pituitary-adrenal axis and the sympathetic nervous system in models of acute stress in domestic farm animals. J Anim Sci 72:1891–1898. 10.2527/1994.7271891x 7928769 10.2527/1994.7271891x

[CR52] Nagorsen DW, Bryant AA, Kerridge D et al (1993) Winter bat records for British Columbia. Northwest Nat 74:61–66

[CR53] Norquay KJO, Martinez-Nuñez F, Dubois JE et al (2013) Long-distance movements of little brown bats (Myotis lucifugus ). J Mammal 94:506–515. 10.1644/12-mamm-a-065.1

[CR54] Palme R (2019) Non-invasive measurement of glucocorticoids: Advances and problems. Physiol Behav 199:229–243. 10.1016/j.physbeh.2018.11.021 30468744 10.1016/j.physbeh.2018.11.021

[CR55] Perry RW, Saugey DA, Crump BG (2010) Winter roosting ecology of silver-haired bats in an Arkansas Forest. Souteastern Naturalist 9:563–572

[CR56] Reeder DAM, Kramer KM (2005) Stress in free-ranging mammals: Integrating physiology, ecology, and natural history. J Mammal 86:225–235. 10.1644/BHE-003.1

[CR57] Reeder DM, Kosteczko NS, Kunz TH, Widmaier EP (2004) Changes in baseline and stress-induced glucocorticoid levels during the active period in free-ranging male and female little brown myotis, Myotis lucifugus (Chiroptera: Vespertilionidae). Gen Comp Endocrinol 136:260–269. 10.1016/j.ygcen.2003.12.020 15028530 10.1016/j.ygcen.2003.12.020

[CR58] Romero LM (2004) Physiological stress in ecology: lessons from biomedical research. Trends Ecol Evol 19:249–255. 10.1016/j.tree.2004.03.008 16701264 10.1016/j.tree.2004.03.008

[CR59] Sandoval-Herrera NI, Mastromonaco GF, Becker DJ, et al (2021) Inter- and intra-specific variation in hair cortisol concentrations of Neotropical bats. Conservat Physiol 9:coab053. 10.1093/conphys/coab053 10.1093/conphys/coab053 PMC8278960 34267922

[CR60] Santymire RM, Ali N, Marinari PE, Livieri TM (2021) Using hair cortisol analysis to understand the biological factors that affect black-footed ferret (*Mustela nigripes* ) stress physiology. Conservat Physiol 9:coab033. 10.1093/conphys/coab033 10.1093/conphys/coab033 PMC8114467 34007452

[CR61] Sapolsky RM, Romero LM, Munck AU (2000) How Do Glucocorticoids influence stress responses? Integrating permissive, suppressive, stimulatory, and preparative actions. Endocr Rev 21:55–89. 10.1210/edrv.21.1.0389 10696570 10.1210/edrv.21.1.0389

[CR62] Sauchyn DJ (1993) Quaternary and late Tertiary landscape evolution in the western Cypress Hills. In: Sauchyn DJ (ed) Quaternary and Late Tertiary Landscapes of Southwestern Saskatchewan and Adjacent Areas. Regina: Canadian Plains Research Centre, Regina, Saskatchewan, pp 46–58

[CR63] Schowalter D, Dorward W, Gonson J (1978) Seasonal occurrence of Silver-haired Bats (Lasionycteris noctivagans) in Alberta and British Columbia. Canadian Field Naturalist 92:288–291

[CR64] Sheriff MJ, Krebs CJ, Boonstra R (2010) The ghosts of predators past: population cycles and the role of maternal programming under fluctuating predation risk. Ecology 91:2983–2994. 10.1890/09-1108.1 21058558 10.1890/09-1108.1

[CR65] Sheriff MJ, Dantzer B, Delehanty B et al (2011) Measuring stress in wildlife: Techniques for quantifying glucocorticoids. Oecologia 166:869–887. 10.1007/s00442-011-1943-y 21344254 10.1007/s00442-011-1943-y

[CR66] Shump KA, Shump AU (1982) Lasiurus cinereus. Mamm Species 185:1–5

[CR67] Sikes RS (2016) 2016 Guidelines of the American Society of Mammalogists for the use of wild mammals in research and education. J Mammal 97:663–688. 10.1093/jmammal/gyw078 29692469 10.1093/jmammal/gyw078 PMC5909806

[CR68] Soper DS (2025) Significance of the Difference between Two Slopes Calculator

[CR69] Stalder T, Kirschbaum C (2012) Analysis of cortisol in hair - State of the art and future directions. Brain Behav Immun 26:1019–1029. 10.1016/j.bbi.2012.02.002 22366690 10.1016/j.bbi.2012.02.002

[CR70] Stead SM, Bădescu I, Boonstra R (2022) Of mammals and milk: how maternal stress affects nursing offspring. Mammal Rev 52:129–147. 10.1111/mam.12267

[CR71] Suter DE, Schwartz NB (1985) Effects of glucocorticoids on secretion of luteinizing hormone and follicle-stimulating hormone by female rat pituitary cells in vitro. Endocrinology 117:849–854. 10.1210/endo-117-3-849 3926469 10.1210/endo-117-3-849

[CR72] Thorup K, Tøttrup AP, Willemoes M et al (2017) Resource tracking within and across continents in long-distance bird migrants. Sci Adv 3:1–11. 10.1126/sciadv.1601360 10.1126/sciadv.1601360 PMC5214581 28070557

[CR73] Tilbrook A (2000) Effects of stress on reproduction in non-rodent mammals: the role of glucocorticoids and sex differences. Rev Reprod 5:105–113. 10.1530/ror.0.0050105 10864855 10.1530/ror.0.0050105

[CR74] Vella CA, Nelson OL, Jansen HT et al (2020) Regulation of metabolism during hibernation in brown bears (Ursus arctos): Involvement of cortisol, PGC-1α and AMPK in adipose tissue and skeletal muscle. Comp Biochem Physiol A Mol Integr Physiol 240:110591. 10.1016/j.cbpa.2019.110591 31669707 10.1016/j.cbpa.2019.110591

[CR75] Weller TJ, Castle KT, Liechti F et al (2016) First direct evidence of long-distance seasonal movements and hibernation in a migratory bat. Sci Rep 6:1–7. 10.1038/srep34585 27698492 10.1038/srep34585 PMC5048302

[CR76] Wikelski M, Cooke SJ (2006) Conservation physiology. Trends Ecol Evol 21:38–46. 10.1016/j.tree.2005.10.018 16701468 10.1016/j.tree.2005.10.018

[CR77] Wilkinson GS, Brunet-Rossinni A (2009) Methods for age estimation and the study of senescence in bats. In: Ecological and behavioral methods for the study of bats. pp 315–325

[CR78] Williams BW, Etter DR, Linden DW et al (2009) Noninvasive hair sampling and genetic tagging of co-distributed fishers and american martens. J Wildl Manag 73:26–34. 10.2193/2007-429

[CR79] Willis CKR, Brigham RM (2005) Physiological and ecological aspects of roost selection by reproductive female hoary bats (Lasiurus cinereus). J Mammal 86:85–94. 10.1007/978-1-4613-0425-8_5

[CR80] Willis CKR, Brigham RM, Geiser F (2006) Deep, prolonged torpor by pregnant, free-ranging bats. Naturwissenschaften 93:80–83. 10.1007/s00114-005-0063-0 16456644 10.1007/s00114-005-0063-0

[CR81] Wingfield JC, Romero LM (2015) Tempests, poxes, predators, and people: stress in wild animals and how they cope. Oxford University Press

[CR82] Wingfield JC, Sapolsky RM (2003) Reproduction and resistance to stress: when and how. J Neuroendocrinology 15:711–724. 10.1046/j.1365-2826.2003.01033.x 12834431 10.1046/j.1365-2826.2003.01033.x

