# Supplementary material for: Differences in fur cortisol levels of three migratory bats
Source: J Comp Physiol B. 2025 Apr 16;195(3):393–404. doi: 10.1007/s00360-025-01609-0 (PMC12289861; doi:10.1007/s00360-025-01609-0)
Supplement: Supplementary file 1 — Supplementary file1 (DOCX 29 KB) [file 360_2025_1609_MOESM1_ESM.docx]

Hoary Bat Hair Cortisol Parallelism (t=1.66, p=0.16, df=5)

Silver-haired Bat Hair Cortisol Parallelism (t=2.18, p=0.08, df=5)

Little Brown Bat Hair Cortisol Parallelism (t=0.02, p=0.98, df=6)
